# Concordance between the Hysteroscopic Diagnosis of Endometrial Hyperplasia and Histopathological Examination

**DOI:** 10.3390/diagnostics9040142

**Published:** 2019-10-07

**Authors:** Pasquale De Franciscis, Gaetano Riemma, Antonio Schiattarella, Luigi Cobellis, Maria Guadagno, Salvatore Giovanni Vitale, Lavinia Mosca, Antonio Cianci, Nicola Colacurci

**Affiliations:** 1 Department of Woman, Child and General and Specialized Surgery, University of Campania “Luigi Vanvitelli”, 80138 Naples, Italy; pasquale.defranciscis@unicampania.it (P.D.F.); aschiattarella@gmail.com (A.S.); luigi.cobellis@unicampania.it (L.C.); maria.guadagno1@hotmail.it (M.G.); laviniamosca@aol.it (L.M.); nicola.colacurci@yahoo.it (N.C.); 2 Unit of Obstetrics & Gynecology, Department of General Surgery and Medical Surgical Specialties, University of Catania, 95123 Catania, Italy; sgvitale@unict.it (S.G.V.); acianci@unict.it (A.C.)

**Keywords:** endometrial hyperplasia, hysteroscopy, postmenopause, endometrial cancer

## Abstract

The goal of this paper is to assess the concordance between the clinical diagnosis of Endometrial Hyperplasia (EH), suspected by senior gynecologists throughout outpatient office hysteroscopy, and the results from histopathological examination, in order to evaluate hysteroscopic accuracy for EH. A prospective cohort study was done at a Tertiary University Hospital. From January to December 2018, we enrolled women with the following criteria: abnormal uterine bleeding in post-menopause and endometrial thickening in pre-or post-menopause. Patients underwent office hysteroscopy with a 5 mm continuous-flow hysteroscope, and endometrial biopsies were taken using miniaturized instruments. Senior operators had to foresee histopathological diagnosis using a questionnaire. Histopathological examination was conducted to confirm the diagnosis. This study was approved by the local ethical and registered in the ClinicalTrials.gov registry (ID no. NCT03917147). In 424 cases, 283 clinical diagnoses of EH were determined by senior surgeons. A histopathological diagnosis was then confirmed in 165 cases (58.3%; *p* = 0.0001). Furthermore, 14 endometrial carcinoma and atypical hyperplasia were found. The sensitivity, positive predictive value, and negative predictive values for EH were, respectively, 90.4, 58.4, and 86.6%. Subdivided by clinical indication, the sensitivity was higher in patients with post-menopause endometrial thickening. The diagnostic accuracy of office hysteroscopy in the diagnosis and prediction of endometrial hyperplasia was high. Senior operators could foresee EHs in more than half the cases.

## 1. Introduction

Hysteroscopy with endometrial biopsy is known to be the gold standard for the diagnosis of malignant and pre-malignant endometrial pathologies and related clinical conditions [1,2]. Hysteroscopy allows the direct visualization of the endometrium and, therefore, the recognition of small and focal anomalies and their targeted biopsy, as opposed to blind sampling techniques, which showed a remarkable incidence of false negatives, especially in cases of focal lesions [3,4,5].

It has been estimated that between 15% and 25% of gynecologists in the United States perform office hysteroscopy and, thanks to an easy-to-improve learning curve, the amount of young and senior operators is still increasing [6]. Nonetheless, hysteroscopy plays a crucial role in the most challenging topics of women’s health, from infertility diagnostic work-up [7,8] to post-menopausal abnormal uterine bleeding and uterine pathologies [9,10]. Currently, available technologies allow one to perform several diagnostic and operative procedures without the need for anesthesia and without any pain or distress for the patient [11,12]. For this reason, the role of dilatation and curettage to assess intrauterine pathologies and abnormal uterine bleeding tends to be no higher than office hysteroscopy [13].

Endometrial Hyperplasia (EH) is considered a heterogeneous pre-neoplastic clinical entity characterized by an abnormal glandular proliferation, with less than half of the tissue area occupied by the stroma. The World Health Organization (WHO) classifies EH in four categories: simple, complex (by the complexity of glandular and disproportion in the gland-to-stroma ratio), simple atypical, and complex atypical (if the cytological atypia is notable) [1,2]

Although hysteroscopy is an excellent tool for the recognition of organic intracavitary diseases, such as submucosal myomas and polyps, the sensitivity and predictive value of hysteroscopic images for endometrial hyperplasia and the correlation of hysteroscopic imaging with anatomopathological data is still debated, with weak and conflicting reports in the literature [2,14,15].

Until now, the morphological criteria used for hysteroscopic diagnosis have been based on the operator’s subjective evaluation and, therefore, are not reproducible. The aim of this study was to evaluate the accuracy of hysteroscopic imaging conducted by senior operators for the diagnosis of endometrial hyperplasia and the correlation of hysteroscopic patterns with the results of histological examination in order to evaluate the accuracy of outpatient office hysteroscopy for EH.

## 2. Materials and Methods

This was meant to be a prospective observational study (Canadian Task Force Classification II-2) on women who underwent outpatient office hysteroscopy at our Hysteroscopy Unit (Obstetric and Gynecological Center, Department of Woman and Child, University of Campania “Luigi Vanvitelli”, Naples, Italy). At the time of the office hysteroscopy, all women gave written consent to use their clinical data for research purposes and approved future contact about research studies.

### 2.1. Inclusion and Exclusion Criteria

The women included in this study were referred by gynecologists from the general outpatient rooms of the institution’s hospital for abnormal uterine bleeding in the post-menopause, ultrasonographic detection of endometrial thickening in pre-menopause and postmenopause, with a follow-up after Tamoxifen-based therapy regimens. Exclusion criteria applied to all the patients were severe urinary symptoms or an age <18 years old.

### 2.2. Outpatient Procedure

Patients underwent the procedure after the menstrual phase (day 6 to 10) of a spontaneous menstrual cycle. All procedures were performed by means of a continuous flow small-diameter hysteroscope with an oval profile (maximum diameter 5 mm, minimum diameter 3.9 mm) (Bettocchi Office Hysteroscope size 5, Karl Storz GmbH and Co., Tuttlingen, Germany), fitted with a 30 degree telescope with a 2.9 mm gauge, using the vaginoscopic approach, without tenaculum and speculum, and using saline solution as a distending medium at 90–100 mm. The Hg pressure was generated by a pneumatic cuff and measured by means of a manometer; the biopsies were performed with the “punch” or “grasp” technique using 5 Fr grasping forceps inserted through the operating channel of the hysteroscope. In the case of small intrauterine pathologies, these were easily removed through a straight bipolar electrode active by an electrosurgical generator (Versapoint II; Gynecare, Ethicon, Cincinnati, USA) used to provide 50 W power to the mildest vapor cutting mode (VC3).

The hysteroscopic diagnosis of hyperplasia was based on one or more of the following findings: (1) focal or diffuse, papillary or polypoid, endometrial thickening, (2) abnormal vascular patterns; (3) evidence of glandular cysts; and (4) abnormal architecture features of the glandular outlets (thickening, irregular gland density, or dilatation). Although no consensus or RCTs showed agreement in describing objective criteria for EH, several trials agreed on the previous morphologic evaluation [16].

The procedures were taken by three senior gynecologists (P.D.F, L.C., and N.C.) whose expertise and skills were equivalent. After every procedure, the surgeons were asked to propose a suggestive histological categorization of the clinical diagnosis by means of a questionnaire, in order to standardize the assessment. The questionnaire was made of a serial number, which identified the biopsy and multiple-choice question. The operators were asked to choose one from the following answers: Benign (including atrophic endometrium, proliferative endometrium, endometrial polyp/s), Endometrial Hyperplasia (simple or complex hyperplasia), or Atypical Hyperplasia/Carcinoma (including atypical endometrial hyperplasia and adenocarcinoma).

Biopsied histological samples were sent for histopathological analysis. The histopathological examination of all the specimens was performed at the Pathology Unit of University of Campania “Luigi Vanvitelli”. Three senior gynecopathologists, all with the same skills and expertise, were addressed to evaluate all the biopsies.

### 2.3. Statistical Analyses

Statistical analysis was conducted using the IBM Statistical Package for Social Sciences (SPSS) v. 19.0. Continuous variables were reported as the average ± SD. Dichotomous data were reported as the absolute number and percentages. Differences in the proportions between the groups were analyzed with the Fisher’s exact and Chi square test, where appropriate. Statistical significance was set as a *p* value of 0.05.

The sensitivity, specificity, positive predictive value, and negative predictive value were calculated with 95% confidence intervals using the Wilson score for binomial proportions. In order to complement the clinical accuracy, we also evaluated the positive and negative likelihood ratios (LR+ and LR−) and pre- and post-test probabilities for EH.

### 2.4. Ethical Approval

This study was approved in date 27/12/2017 by the local ethical committee of the University of Campania with protocol number 592-27/12/2017, registered into the ClinicalTrials.gov registry with ID NCT03917147; it was carried out in accordance with principles of the Helsinki Declaration of 1975, using routine clinical practice procedures usually performed during the hysteroscopic procedure. Such procedures did not involve any additional risk to the patients, and all the medical decisions concerning individual patients were not affected by the study. The confidentiality of the participants was maintained during the data analysis.

## 3. Results

The main characteristics of the women enrolled in our study are described in Table 1.

Four hundred and twenty-four women were prospectively enrolled and subdivided by clinical indication: 92 for post-menopause AUB, 225 for post-menopause endometrial thickening, and 107 for pre-menopause endometrial thickening.

The hysteroscopic findings and concordance to histopathology are described in Table 2.

Considering the whole number of patients, in 58.3% (165/283; *p* = 0.0001) of the cases, the operators successfully predicted the diagnosis of endometrial hyperplasia; 85.8% (109/127; *p* = 0.0001) of the hysteroscopic impressions were concordant with histopathology when we discovered a benign disease or a normal endometrium.

When women were stratified by indication, as shown in Table 3, the highest concordance was found when diagnosing EH in women with pre-menopausal endometrial thickening (69.8%, *p* = 0.0076), and the lowest was found in post-menopause (51.6%, *p* = 0.0059).

In the case of a normal endometrial pattern or a benign pathology, the correlated histopathological results were high (from 80.8% in post-menopause AUB to 95.7% in post-menopause endometrial thickening). No cases of inadequate sampling were found.

The calculated percentages of the sensibility, positive and negative predictive value, and specificity of the hysteroscopic technique (as compared to the histopathological examination) for the endometrial hyperplasia are shown in Table 4.

The overall sensibility for EH was high (90.4%), with a notable NPV of 86.6%. Although misdiagnosed EH led to the discovery that specificity and PPV were low–moderate (respectively, 48.9 and 44.2%). The highest sensitivity could be found in women with post-menopause endometrial thickening (97.9%). The NPV for this group was 95.9%.

The LR+, LR−, and pre- and post-test probability for hysteroscopy identifying EH are also shown in Table 4. It is notable that LR− was generally around 0.1, especially in patients with postmenopausal endometrial thickening (0.05)

A reasonably low number of findings concerned endometrial adenocarcinoma or atypical endometrial hyperplasia (14/435). In the post-menopausal AUB group, we discovered one case of complex atypical hyperplasia and one grade-1 endometrioid adenocarcinoma. In women with post-menopausal endometrial thickening, ten cases of complex atypical endometrial hyperplasia, one grade-1, and one grade-2 endometrioid adenocarcinoma were found. The hysteroscopic and histopathological examinations were concordant in every case (100%).

## 4. Discussion

Our data demonstrate that hysteroscopic examination of EH shows high sensitivity and a negative predictive value with a low positive predictive value. When stratified by indication, the sensitivity remained high, and the positive predictive value improved, especially in post-menopause endometrial thickening patients [17,18].

In a 2014 meta-analysis, Gkrozou et al. reported that the diagnostic accuracy of hysteroscopy is high for endometrial cancer, polyps, and submucous myomas, but only moderate for endometrial hyperplasia [19]. The lack of objective and standardized criteria for endometrial hyperplasia leads to the use of hysteroscopic biopsies for all the women for whom the hysteroscopic vision suspects such pathologies [20]. This practice is likely associated with more biopsies than necessary. However, endometrial hyperplasia is frequently a widespread pathological process, with a combination of the single elementary findings described above.

A 2018 randomized clinical trial showed that performing a hysteroscopy first ensures a better image, whereas a biopsy yields an adequate tissue sample with fewer attempts [21]. In the current literature, there are no systematic correlation data between the hysteroscopic features of a single hysteroscopic elementary lesion and the diagnosis of endometrial hyperplasia. Since the accuracy of hysteroscopy on the diagnosis of endometrial hyperplasia has never been evaluated in randomized controlled trials, there is no universal and reproducible morphological hysteroscopic definition of endometrial hyperplasia.

For example, based on the evidence available in the literature, the architectural distortion of geometry, both structural (abnormal spacing and/or dilatation of the glandular orifices) and hyperchromatic (glandular openings that are yellowish-white in color), represents a finding that is highly suggestive of endometrial hyperplasia and should be sampled for histopathological studies. Nonetheless, this dilatation of the glandular orifices is also common in other benign patterns (such as senile cystic atrophy of the endometrium or tamoxifen-associated atrophy), making it difficult to diagnose endometrial hyperplasia over other diseases [22]. Therefore, the current state of knowledge does not allow to establish a standardized morphological target to address a direct biopsy, which, instead, often relies on the operator’s experience [23,24,25].

However, the high rates of senior operators in detecting a normal endometrium or benign pathologies and the high NPV for endometrial hyperplasia could be useful in avoiding unnecessary biopsies, especially in patients with low-risk factors [26].

Van Hanegem et al. reported that in women with postmenopausal bleeding, the sensitivity of endometrial sampling to detect endometrial cancer, and especially atypical hyperplasia and endometrial disease, including endometrial polyps, is lower than previously thought. However, our data suggest that, for trained surgeons, outpatient office hysteroscopy with guided biopsies still results in high sensitivity when patients are categorized by their indication to hysteroscopy [27]. The clinical indication of hysteroscopy significantly influences not only the attitude of the operator to performing an endometrial biopsy, but also its real clinical usefulness. Indeed, in women with a strong clinical suspicion of endometrial pathology (postmenopausal AUB, suspected ultrasound of endometrial thickening, tamoxifen therapy, peri-menopausal women under estrogen therapy) [28], we did not observe an excess of biopsies, with a significant concordance between hysteroscopic imaging and anatomopathological data. On the other hand, in women with premenopausal AUB, we observed an excess of biopsies not consistent with anomalous anatomopathological data.

A frequent error is the hysteroscopic finding of the normal endometrium, rather than the hyperplastic endometrium, which is then diagnosed histologically [26,29,30]. These findings are likely associated with an intrinsic bias of the operator, which is considered a reasonable functional condition that determines the presence of homogeneous endometrial thickening with a regular surface. Differences in the definition of the normal endometrium [31] are likely the cause of the differences in sensitivity reported in the literature, which ranged from 16% to 98% [17,25]. From this perspective, a noticeable increase in sensitivity could be achieved by conforming to the stringent criteria of Loffer, who considers a uniformly thin endometrium normal without anatomical distortions of the endometrial cavity with good visualization [32]. The limits of the current hysteroscopic criteria in predicting endometrial hyperplasia also raise a question about the most reliable method to engage in endometrial sampling for histological evaluation. There is no doubt that hysteroscopy and targeted biopsy represent a diagnostic evolution of blind techniques, such as pipelle, in the identification of focal endometrial pathologies, including those of a hyperplastic type [3,4,27,33]. At present, blind biopsies commonly do not allow any focal lesions to be assessed, but some authors suggest that biopsies under hysteroscopic guidance could be significantly improved by the use of blind sampling, especially when the hyperplastic process is widespread [21].

In the absence of valuable hysteroscopic elements that suggest hyperplasia and its severity, it is hypothesized that “eye-directed” biopsies might leave out atypical lesions or carcinomas in the initial phase. These lesions or carcinomas often coexist with less severe forms of hyperplasia [3,4]. There are no publications that respond to these concerns. Instead, we are waiting for a comparative study on the recognition of hyperplasia with a blind biopsy or under hysteroscopic guidance. In our opinion, we should modulate biopsies according to the hysteroscopic framework, even in cases with minimal suspicion, even if this attitude can increase the number of “superfluous” biopsies and thus reduce the specificity of the examination. There is no doubt about the need to do a biopsy when there are anomalous focal lengths in a thin endometrium [34]. Nonetheless, at the moment, in relation to the available instruments (optics, distension media, biopsy forceps) and to the morphological criteria used, it is not possible to hypothetically reduce the number of biopsies to increase the specificity of the examination, because of the risk of disregarding hyperplasia pictures with atypia. Because of this problem, it is necessary to perform biopsies for all hysteroscopies that show an irregular or thickened endometrium, in which hyperplasia cannot be excluded.

## 5. Conclusions

Adopting the current morphological criteria, we found that the accuracy of hysteroscopy in the diagnosis and the prediction of endometrial hyperplasia is high. A low negative likelihood ratio, combined with a high negative predictive value, shows that a clinical hysteroscopic diagnosis could be beneficial in excluding endometrial hyperplasia, especially when post-menopausal endometrial thickening is observed ultrasonographically. It is, therefore, necessary to improve not only the predictive morphological hysteroscopic criteria of endometrial hyperplasia but also a wider consensus is needed to standardize the clinical criteria for this type of pre-neoplastic disease.

## Figures and Tables

**Table 1:** The baseline characteristics of the women included in the study. Values are given as the mean ± Standard Deviation, as appropriate.

	Total	Post Menopause Abnormal Uterine Bleeding (AUB)	Post Menopause Endometrial Thickening	Pre Menopause Endometrial Thickening
Patients (*n*)	424	92	225	107
Age (years)	49.6 ± 4.2	51.0 ± 5.9	57.4 ± 3.2	41.3 ± 3.4
Weight (Kg)	67.1 ± 5.2	69.4 ± 3.4	68.3 ± 4.5	64.1 ± 4.5
Body-Mass Index (Kg/m^2^)	23.9 ± 4.3	24.2 ± 6.1	24.1 ± 5.6	24.7 ± 5.9

**Table 2:** Summary of the correlations between hysteroscopic findings and histopathologic examination. The data are expressed as frequencies (percentages).

Diagnosis	Hysteroscopy	Histopathology		*p* Value
		Correlates	Does Not Correlate	
Normal/Benign	127	109 (85.8)	18 (14.1)	0.0001
Endometrial Hyperplasia	283	165 (58.3)	118 (41.7)	0.0001
Atypical Hyperplasia/Carcinoma	14	14 (100)	0	0.0065
TOTAL	424	288 (67.9)	136 (32.1)	0.0001

**Table 3:** Correlation between the hysteroscopic findings and the histopathology for each group.

Diagnosis	Hysteroscopy	Histopathology		*p* Value
		Correlates	Does Not Correlate	
**AUB in Post-Menopause**				
Normal/Benign	26	21 (80.8)	5 (19.2)	0.0176
Endometrial Hyperplasia	64	33 (51.6)	31 (48.4)	0.0059
Atypical Hyperplasia/Carcinoma	2	2 (100)	0	0.5184
TOTAL	92	56 (60.9)	36 (39.1)	0.0150
**Endometrial Thickening in Post-Menopause**				
Normal/Benign	47	45 (95.7)	2 (4.3)	0.0001
Endometrial Hyperplasia	166	94 (56.6)	72 (43.4)	0.0001
Atypical Hyperplasia/Carcinoma	12	12 (100)	0	0.0099
TOTAL	225	151 (67.1)	74 (32.9)	0.0001
**Endometrial Thickening in Pre-menopause**				
Normal/Benign	54	49 (90.7)	5 (9.3)	0.0076
Endometrial Hyperplasia	53	37 (69.8)	16 (30.2)	0.0076
Atypical Hyperplasia/Carcinoma	0	0	0	NA
TOTAL	107	86 (80.4)	21 (19.6)	0.0080

Data are expressed as the frequencies (percentages). NA = not applicable.

**Table 4:** Accuracy of the office hysteroscopy for endometrial hyperplasia, with histopathology as a reference.

	Total	AUB in Postmenopause	Endometrial Thickening in Postmenopause	Endometrial Thickening in Premenopause
Sensitivity	90.4 (87.1–93.0)	86.8 (77.8–92.7)	97.9 (94.7–93.3)	88.1 (80.1–93.3)
Specificity	48.9 (44.1–53.8)	42.6 (32.5–53.3)	39.5 (33.0–46.4)	75.4 (65.9–83.0)
Pre-test	44.2 (53.6–63.1)	41.3 (31.3–52.1)	44.7 (37.9–51.6)	39.3 (30.1–49.2)
PPV	58.4 (53.6–63.1)	51.6 (41.0–62.0)	56.6 (49.7–63.3)	69.8 (60.1–78.1)
NPV	86.6 (82.9–89.6)	82.1 (72.5–89.1)	95.9 (92.1–98.0)	90.7 (83.2–95.2)
LR+	1.77 (1.54–2.03)	1.51 (1.01–2.11)	1.59 (1.26–2.00)	3.58 (3.01–4.27)
LR−	0.14 (0.11–0.19)	0.30 (0.22–0.43)	0.05(0.04–0.06)	0.15 (0.13–0.18)
Post test (+)	0.58 (0.52–0.64)	0.51 (0.37–0.65)	0.56 (0.47–0.65)	0.69 (0.58–0.79)
Post test (−)	0.13 (0.04–0.22)	0.17 (0.01–0.37)	0.04 (0.02–0.16)	0.01 (0.01–0.21)

Values are given as the percentage and confidence intervals. Pre-test: pre-test probability; PPV: positive predictive value; NPV: negative predictive value; LR+: positive likelihood ratio; LR−: negative likelihood ratio; post test (+): positive post test probability; post test (−): negative post test probability.

## References

[1] Litta P., Merlin F., Saccardi C., Pozzan C., Sacco G., Fracas M., Capobianco G., Dessole S. (2005). Role of hysteroscopy with endometrial biopsy to rule out endometrial cancer in postmenopausal women with abnormal uterine bleeding. Maturitas.

[2] Gubbini G., Filoni M., Linsalata I., Stagnozzi R., Stefanetti M., Marabini A. (1998). The role of hysteroscopy in the diagnosis and follow-up of endometrial hyperplasia. Minerva Ginecol..

[3] Abdelazim I.A., Abdelrazak K.M., Elbiaa A.A., Al-Kadi M., Yehia A.H. (2015). Accuracy of endometrial sampling compared to conventional dilatation and curettage in women with abnormal uterine bleeding. Arch. Gynecol. Obstet..

[4] Bedner R., Rzepka-Gorska I. (2007). Hysteroscopy with directed biopsy versus dilatation and curettage for the diagnosis of endometrial hyperplasia and cancer in perimenopausal women. Eur. J. Gynaecol. Oncol..

[5] Lee D.O., Jung M.H., Kim H.Y. (2011). Prospective comparison of biopsy results from curettage and hysteroscopy in postmenopausal uterine bleeding. J. Obstet. Gynaecol. Res..

[6] Vitale S.G., Capriglione S., Zito G., Lopez S., Gulino F.A., Di Guardo F., Vitagliano A., Noventa M., La Rosa V.L., Sapia F. (2019). Management of endometrial, ovarian and cervical cancer in the elderly: Current approach to a challenging condition. Arch. Gynecol. Obstet..

[7] Colacurci N., Caprio F., La Verde E., Trotta C., Ianniello R., Mele D., De Franciscis P. (2014). Sequential protocol with urinary-FSH/recombinant-FSH versus standard protocol with recombinant-FSH in women of advanced age undergoing IVF. Gynecol. Endocrinol..

[8] Vitale S.G., Padula F., Gulino F.A. (2015). Management of uterine fibroids in pregnancy: Recent trends. Curr. Opin. Obstet. Gynecol..

[9] Lagana A.S., Vitale S.G., Stojanovska L., Lambrinoudaki I., Apostolopoulos V., Chiofalo B., Rizzo L., Basile F. (2018). Preliminary results of a single-arm pilot study to assess the safety and efficacy of visnadine, prenylflavonoids and bovine colostrum in postmenopausal sexually active women affected by vulvovaginal atrophy. Maturitas.

[10] Torella M., Del Deo F., Grimaldi A., Iervolino S.A., Pezzella M., Tammaro C., Gallo P., Rappa C., De Franciscis P., Colacurci N. (2016). Efficacy of an orally administered combination of hyaluronic acid, chondroitin sulfate, curcumin and quercetin for the prevention of recurrent urinary tract infections in postmenopausal women. Eur. J. Obstet. Gynecol. Reprod. Biol..

[11] Lagana A.S., Vergara D., Favilli A., La Rosa V.L., Tinelli A., Gerli S., Noventa M., Vitagliano A., Triolo O., Rapisarda A.M.C. (2017). Epigenetic and genetic landscape of uterine leiomyomas: A current view over a common gynecological disease. Arch. Gynecol. Obstet..

[12] Vitale S.G., Sapia F., Rapisarda A.M.C., Valenti G., Santangelo F., Rossetti D., Chiofalo B., Sarpietro G., La Rosa V.L., Triolo O. (2017). Hysteroscopic Morcellation of Submucous Myomas: A Systematic Review. BioMed Res. Int..

[13] Agostini A., Collette E., Provansal M., Estrade J.P., Blanc B., Gamerre M. (2008). Good practice and accuracy of office hysteroscopy and endometrial biopsy. J. Gynecol. Obstet. Biol. Reprod..

[14] Gan D.E., Jawan R.A., Moy F.M. (2013). Concordance between hysteroscopic impression and endometrial histopathological diagnosis. Prev. Med..

[15] Lasmar R.B., Dias R., Barrozo P.R., Oliveira M.A., Coutinho Eda S., da Rosa D.B. (2008). Prevalence of hysteroscopic findings and histologic diagnoses in patients with abnormal uterine bleeding. Fertil. Steril..

[16] Clark T.J., Voit D., Gupta J.K., Hyde C., Song F., Khan K.S. (2002). Accuracy of hysteroscopy in the diagnosis of endometrial cancer and hyperplasia: A systematic quantitative review. JAMA.

[17] Clarke M.A., Long B.J., Del Mar Morillo A., Arbyn M., Bakkum-Gamez J.N., Wentzensen N. (2018). Association of Endometrial Cancer Risk with Postmenopausal Bleeding in Women: A Systematic Review and Meta-analysis. JAMA Intern. Med..

[18] Trojano G., Damiani G.R., Casavola V.C., Loiacono R., Malvasi A., Pellegrino A., Siciliano V., Cicinelli E., Salerno M.G., Battini L. (2018). The Role of Hysteroscopy in Evaluating Postmenopausal Asymptomatic Women with Thickened Endometrium. Gynecol. Minim. Invasive Ther..

[19] Gkrozou F., Dimakopoulos G., Vrekoussis T., Lavasidis L., Koutlas A., Navrozoglou I., Stefos T., Paschopoulos M. (2015). Hysteroscopy in women with abnormal uterine bleeding: A meta-analysis on four major endometrial pathologies. Arch. Gynecol. Obstet..

[20] Bourdel N., Chauvet P., Tognazza E., Pereira B., Botchorishvili R., Canis M. (2016). Sampling in Atypical Endometrial Hyperplasia: Which Method Results in the Lowest Underestimation of Endometrial Cancer? A Systematic Review and Meta-analysis. J. Minim. Invasive Gynecol..

[21] Sarkar P., Mikhail E., Schickler R., Plosker S., Imudia A.N. (2017). Optimal Order of Successive Office Hysteroscopy and Endometrial Biopsy for the Evaluation of Abnormal Uterine Bleeding: A Randomized Controlled Trial. Obstet. Gynecol..

[22] Nappi C., Di Spiezio Sardo A. (2014). State-of-the-Art Hysteroscopic Approaches to Pathologies of the Genital Tract.

[23] Ghoubara A., Sundar S., Ewies A.A.A. (2018). Predictors of malignancy in endometrial polyps: Study of 421 women with postmenopausal bleeding. Climacteric.

[24] Trimble C.L., Kauderer J., Zaino R., Silverberg S., Lim P.C., Burke J.J., Alberts D., Curtin J. (2006). Concurrent endometrial carcinoma in women with a biopsy diagnosis of atypical endometrial hyperplasia: A Gynecologic Oncology Group study. Cancer.

[25] Trimble C.L., Method M., Leitao M., Lu K., Ioffe O., Hampton M., Higgins R., Zaino R., Mutter G.L. (2012). Society of Gynecologic Oncology Clinical Practice Committee. Management of endometrial precancers. Obstet. Gynecol..

[26] Siciliano R.A., Mazzeo M.F., Spada V., Facchiano A., d’Acierno A., Stocchero M., De Franciscis P., Colacurci N., Sannolo N., Miraglia N. (2014). Rapid peptidomic profiling of peritoneal fluid by MALDI-TOF mass spectrometry for the identification of biomarkers of endometriosis. Gynecol. Endocrinol..

[27] Van Hanegem N., Prins M.M., Bongers M.Y., Opmeer B.C., Sahota D.S., Mol B.W., Timmermans A. (2016). The accuracy of endometrial sampling in women with postmenopausal bleeding: A systematic review and meta-analysis. Eur. J. Obstet. Gynecol. Reprod. Biol..

[28] De Franciscis P., Cobellis L., Fornaro F., Sepe E., Torella M., Colacurci N. (2007). Low-dose hormone therapy in the perimenopause. Int. J. Gynaecol. Obstet..

[29] Kurt S., Demirtas O., Kopuz A., Beyan E., Demirtas G., Besler A., Altinboga O. (2012). Evaluation of the histopathological diagnosis of patients preoperatively diagnosed with atypical endometrial hyperplasia after hysterectomy. Eur. J. Gynaecol. Oncol..

[30] Vitale S.G., Capriglione S., Peterlunger I., La Rosa V.L., Vitagliano A., Noventa M., Valenti G., Sapia F., Angioli R., Lopez S. (2018). The Role of Oxidative Stress and Membrane Transport Systems during Endometriosis: A Fresh Look at a Busy Corner. Oxid. Med. Cell. Longev..

[31] Campitiello M.R., De Franciscis P., Mele D., Izzo G., Sinisi A., Delrio G., Colacurci N. (2011). Endometrial LGR7 expression during menstrual cycle. Fertil. Steril..

[32] Loffer F.D. (1989). Hysteroscopy with selective endometrial sampling compared with D&C for abnormal uterine bleeding: The value of a negative hysteroscopic view. Obstet. Gynecol..

[33] Machado F., Moreno J., Carazo M., Leon J., Fiol G., Serna R. (2003). Accuracy of endometrial biopsy with the Cornier pipelle for diagnosis of endometrial cancer and atypical hyperplasia. Eur. J. Gynaecol. Oncol..

[34] Socolov D., Socolov R., Rugina V., Gabia R.O., Caraueanu D.M., Lupascu I.A., Carauleanu A. (2016). A Thin and Regular Endometrium in Endometrial Cancer—Endovaginal Ultrasound Assessment. Rev. Med. Chir. Soc. Med. Nat. Iasi.

